# A foodborne outbreak caused by atypical enteropathogenic *Escherichia coli* O45:H15 in the Kinki region of Japan

**DOI:** 10.1128/aem.00123-25

**Published:** 2025-05-27

**Authors:** Etsuko Saito, Kenichi Ogita, Tetsuya Harada, Yuki Wakabayashi, Takako Yagi, Takahiro Yamaguchi, Tomohiro Oshibe, Tetsuhiko Oooka, Takao Kawai

**Affiliations:** 1 Division of Infectious Disease, Hyogo Prefectural Institute of Public Health Science, Kakogawa, Hyogo, Japan; 2 Bacteriology Section, Division of Microbiology, Osaka Institute of Public Health 91397 , Osaka, Osaka, Japan; 3 Food Sanitation and Pharmaceutical Affairs Division, Hyogo Prefectural Government Kitaharima District Administration Office Kato Health & Welfare Office, Kato, Hyogo, Japan; INRS Armand-Frappier Sante Biotechnologie Research Centre, Laval, Quebec, Canada

**Keywords:** atypical EPEC, foodborne outbreak, whole genome sequencing, type 3 secretion system effector

## Abstract

Atypical enteropathogenic *Escherichia coli* (aEPEC) mainly causes sporadic diarrhea and occasional outbreaks. However, the genetic determinant of aEPEC causing large outbreaks is still unknown. In June 2022, 171 of 934 people presented with diarrhea and abdominal pain after eating a lunch box in the Kinki region of Japan. We investigated 44 fecal samples from persons who ate the cuisine and isolated enteropathogenic *Escherichia coli* (EPEC) serotype O45:H15 from 38 of them. The same pathogen was also isolated from the feces of two employees and a leftover sample (mashed tofu salad with spinach). Pulsed-field gel electrophoresis and whole genome sequencing supported the clonality of the isolates. The isolates were negative for *bfpA*, encoding the bundle-forming pilus, and were accordingly identified as aEPEC. Whole genome sequencing revealed the presence of a plasmid-encoded type 3 secretion system effector gene, *espT*, involving the invasive phenotype of EPEC. Finally, we concluded that this was a foodborne outbreak caused by aEPEC O45:H15. Since the food poisoning case caused by aEPEC O45:H15 harboring *espT* has not been reported previously, the current study broadens our understanding of aEPEC food poisoning and its genetic background.

**IMPORTANCE:**

aEPEC causes diarrhea in humans, despite the reported asymptomatic carriers of aEPEC worldwide. Several outbreaks caused by aEPEC also support that this pathogen is a diarrheagenic agent; however, the genetic determinant of aEPEC causing large outbreaks is still unclear. In 2022, a large foodborne outbreak by aEPEC O45:H15 affected more than 170 people in the Kinki region of Japan. We sequenced the whole genomes of the etiological agents and identified a potential virulent plasmid carrying *espT*, which is a virulence factor of aEPEC O111 that caused diarrhea in more than 600 people in Finland. Our data strengthen the importance of *espT* as a virulence factor of aEPEC outbreaks.

## INTRODUCTION

*Escherichia coli* is a commensal bacteria of the human intestine. However, some groups of *E. coli* strains called diarrheagenic *Escherichia coli* (DEC) cause enteric disease in humans. To date, at least six pathotypes have been included in DEC: enteropathogenic *Escherichia coli* (EPEC), enteroinvasive *E. coli*, enterotoxigenic *E. coli*, enterohaemorrhagic *E. coli* (EHEC), enteroaggregative *E. coli*, and diffusely adherent *E. coli* (1). While DEC is classified by their attachment pattern to host cells or repertoire of virulence factor genes, new types of DEC or hybrid types have recently emerged (2, 3).

Among DEC pathotypes, the first described pathotype of diarrheagenic *E. coli* was EPEC (1). EPEC is a leading cause of infant diarrhea, mainly in developing countries. Several studies have reported a striking age distribution of EPEC pathogenesis; in particular, children aged younger than 2 years are vulnerable, whereas adults are not (4). Historically, the laboratory identification of EPEC was based on the O and H serotyping of isolates. In his review, Levine described 14 O serogroups (O26, O55, O86, O111, O119, O125, O126, O127, O128ab, O142, O18, O44, O112, and O114) associated with the EPEC pathotype (5). These serogroups have also been referred to as the “classical” O serogroups (6). Subsequently, along with progress in understanding the molecular mechanisms of EPEC pathogenicity, the laboratory identification of EPEC had shifted from serological methods to genetic or phenotypical methods. This resulted in the discovery of various O serogroups of EPEC strains, including non-classical O serogroups, with divergent genetic backgrounds (6).

The primary virulence factor of EPEC is the *eae* gene encoding an outer membrane protein called intimin, which mediates intimate attachment to host intestinal epithelial cells in the formation of attaching and effacing (A/E) lesions. Intimin interacts with the translocated intimin receptor, which is secreted to the host cell by the type 3 secretion system (T3SS), forming A/E lesions with the cooperation of other effector proteins (7). The *eae* gene, several effector genes including *tir*, and genes encoding components of T3SS are all located on a large pathogenicity island called the locus of enterocyte effacement (LEE), albeit that effectors encoded outside the LEE region (non-LEE effectors) have also been reported (7, 8).

Another important virulence factor is bundle-forming pilus (BFP). Although BFP is not necessary for the formation of A/E lesions, BFP contributes to the initial attachment of EPEC to host cells (4). The *bfp* operon, including *bfpA* encoding bundlin, a major pilus subunit, is carried on a plasmid called the EPEC adherence factor (EAF) plasmid. EPEC strains are divided into two groups based on the presence or absence of the EAF plasmid: typical EPEC (tEPEC) and atypical EPEC (aEPEC) (4). tEPEC is defined as both *eae* and *bfp* positive, whereas aEPEC is *eae* positive but *bfp* negative.

Although the pathogenicity of tEPEC has been confirmed by volunteer studies and epidemiological studies, that of aEPEC is controversial (9). A volunteer study by Levine et al. revealed that the aEPEC O128:H2 strain did not cause diarrhea in adult volunteers (10). Another volunteer study revealed that tEPEC O127:H6 without EAF plasmid was less virulent and thereby implied the importance of *bfp* to the pathogenesis of this strain (11). Some epidemiological studies have demonstrated an association of aEPEC with diarrhea, whereas others reported no difference in the isolation ratio of aEPEC between diarrheal patients and controls (6).

Nevertheless, numerous reports of outbreaks by aEPEC have confirmed this pathogen as a diarrheagenic agent. In 1987, aEPEC O111:B4 caused a large outbreak which involved 611 pupils and 39 adults in Finland (12). A large foodborne outbreak by aEPEC O39:HNM occurred in 1991, involving more than 100 patients (13). In Japan, a waterborne outbreak by aEPEC ONT:H45 was reported (14). Additionally, atypical EPEC O157:H45 and O127a:K63 were also reported as the causative agents of foodborne outbreaks in 2013 and 2010, respectively (15, 16). These various findings suggest that some, but not all, aEPEC strains are virulent to humans. Nevertheless, the genetic background of these outbreak-related strains is poorly understood, except for a few cases (16, 17).

In June 2022, a large foodborne outbreak occurred following the consumption of a contaminated boxed lunch in the Kinki region of Japan. Upon investigation, we identified aEPEC O45:H15 as an etiological agent of the outbreak. Here, we report details of the epidemiological and bacterial investigation of this outbreak. Furthermore, we conducted whole genome sequencing to characterize the genomic background of the causative agent of this outbreak.

## RESULTS

### Overview of the food poisoning case and epidemiological investigations

**Fig 1:**
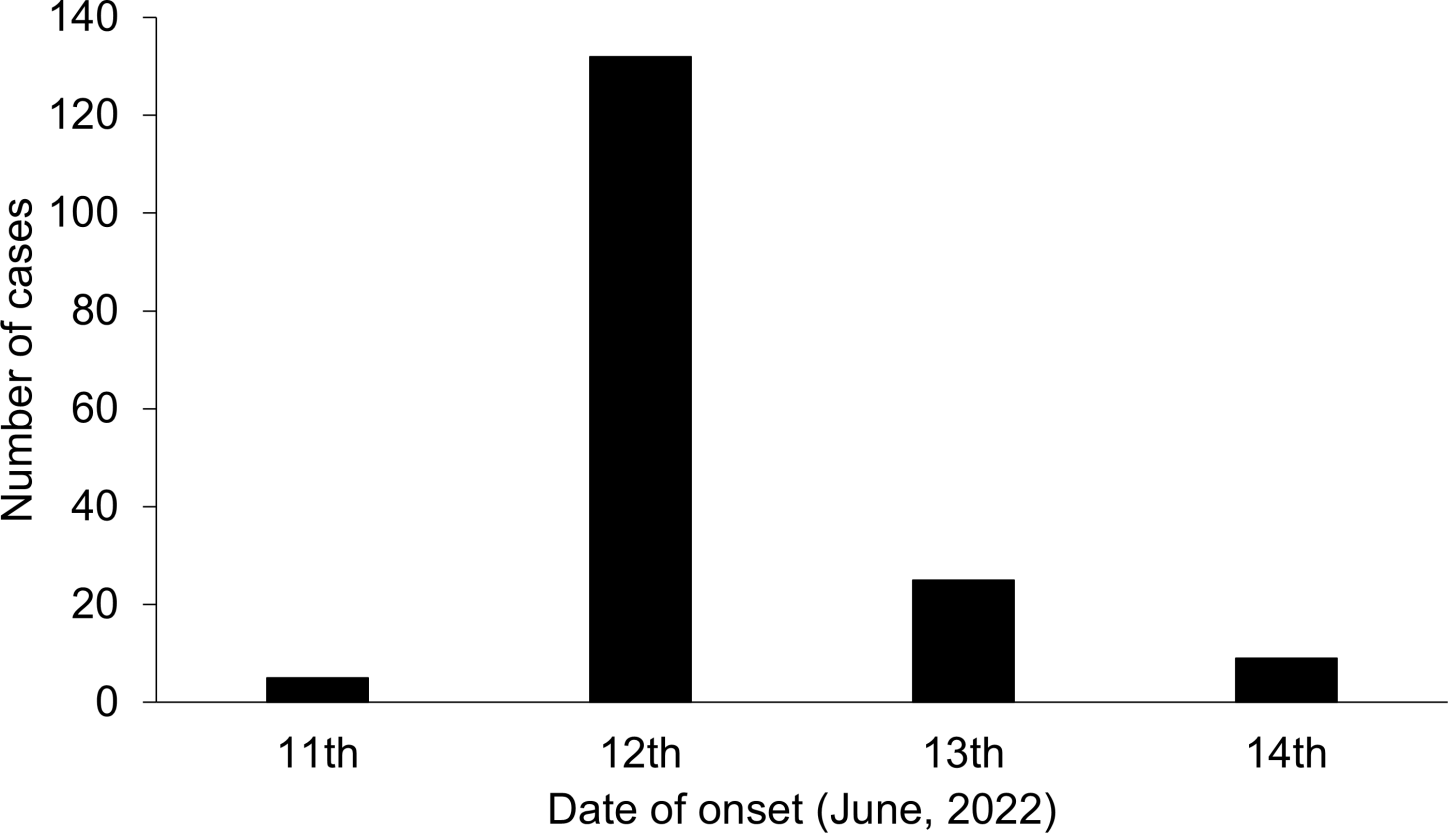
Epidemic curve of the outbreak.

On 12 June 2022, a public health department in Hyogo Prefecture, Japan, was notified of complaints of gastrointestinal disease among those who ate a lunch box made by a particular restaurant. The standard questionnaires administered by health agencies revealed that 171 of 251 patrons who ate a lunch box made by the restaurant between 11 and 13 June developed clinical symptoms (Fig. 1).

Among patients, 87.1% experienced diarrhea, 84.2% abdominal pain, 42.7% fever, 42.1% headache, 35.7% malaise, 25.2% chills, 19.3% nausea, 12.9% tenesmus, 5.3% belching, and 3.5% vomiting. The median incubation period was 15.5 h (range, 1–43 h). The median age of all patients was 17 years (range, 5–84 years), and 59.1% were female (Table 1).

**TABLE 1:** Age distribution of patients who fell ill during the investigated outbreak

		No. of patients in age group:										Total
		0–4 yr	5–9 yr	10–14 yr	15–19 yr	20–29 yr	30–39 yr	40–49 yr	50–59 yr	60–69 yr	≥70 yr	
Male		0	3	1	38	3	5	5	9	3	3	70
Female		0	0	0	84	4	2	3	3	1	4	101

### Laboratory investigations and bacterial identification

The Osaka Institute of Public Health collected 20 fecal samples from patients to examine the presence of foodborne pathogens. The Hyogo Prefectural Institute of Public Health Science and Hyogo Prefectural Health and Welfare Offices obtained 34 fecal samples from 24 additional patients and 10 employees, including food handlers; 10 environmental swabs from the kitchen; and 6 available food samples served to patients to explore for the possible presence of foodborne pathogens.

We identified 41 EPEC isolates from 38 patients (86.4%), 2 employees, and 1 food sample (mashed tofu salad with spinach) in total. We initially tried to identify the O-antigen of the isolates using a commercially available antiserum kit provided by a Japanese supplier, Denka (Tokyo, Japan). However, the isolates were not agglutinated with any of the 50 types of O-antigen-antiserum included in the kit. H serotyping was also performed with antiserum provided by Denka; however, the H-antigen of the isolates was untypable with the commercial kit. Therefore, we used genotyping PCR to identify the O-antigen as well as the H-antigen of the isolates. All 41 EPEC were identified by the PCR as an identical genotype, Og45:Hg15. We further performed serotyping with antiserum supplied by the Serum Status Institute and confirmed the isolates were agglutinated with O45- and H15-antisera.

All 41 EPEC O45:H15 isolates were subjected to a PCR method to detect *bfpA*. All isolates were negative for *bfpA* and were accordingly categorized as aEPEC. Antimicrobial susceptibility testing revealed that 40 of the EPEC serotype O45:H15 isolates were not resistant to any of the antimicrobials used in this research, whereas one isolate from a patient was resistant to chloramphenicol, streptomycin, and tetracycline.

**Fig 2:**
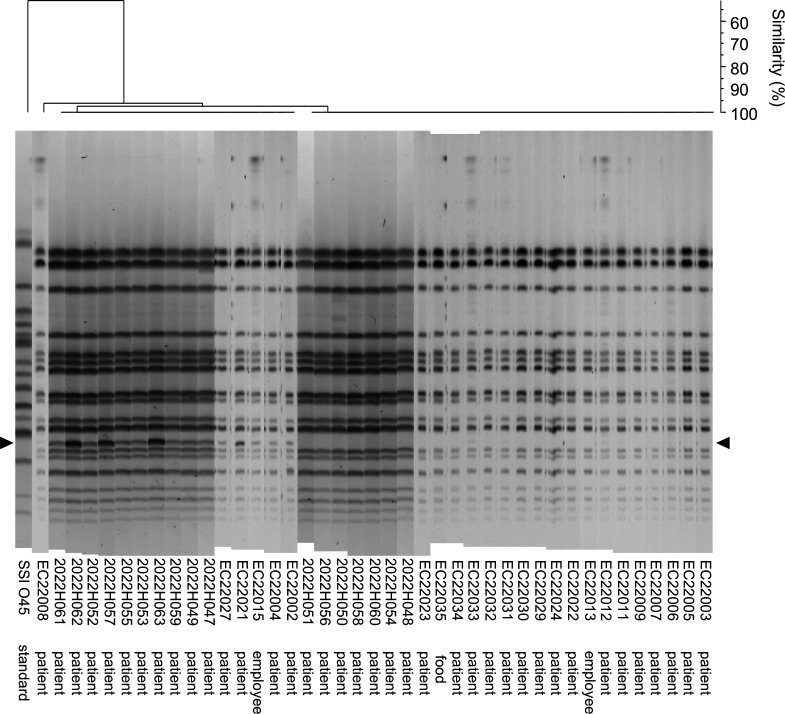
Clustering analysis of the EPEC O45 isolates by PFGE band patterns. Black arrowheads indicate a band of around 90 kb. Dice coefficient similarity with the UPGMA dendrogram is shown above the PFGE gel image.

We then performed pulsed-field gel electrophoresis (PFGE) to confirm the clonality of the isolates. PFGE divided the 41 isolates into two major groups by the presence of a band with a size of around 90 kb (Fig. 2). Although several variations appeared on band patterns, all isolates recovered from patients, employees, and food samples were similar within two band differences. Cluster analysis of the isolates resulted in more than 90% similarity of PFGE patterns. We also sequenced the whole genomes of seven representative strains (2022H047, 2022H048, EC22002, EC22005, EC22013, EC22015, and EC22035) that were isolated from different sources in two independent laboratories, since PFGE has low discrimination power compared to whole genome sequencing. Whole genome sequencing of the isolates also supported the results of PFGE: no single-nucleotide variants (SNVs) were found on the chromosomes of the seven sequenced strains. This result was also confirmed with Snippy and CFSAN SNV-calling pipelines. From these results, we concluded that the causative pathogen of the foodborne diarrheal outbreak under investigation was a specific clone of aEPEC serotype O45:H15. This conclusion was supported by the fact that no other foodborne bacteria or viruses apart from aEPEC O45:H15 were detected in this outbreak.

### Whole genome sequencing of the causative agent

**Fig 3:**
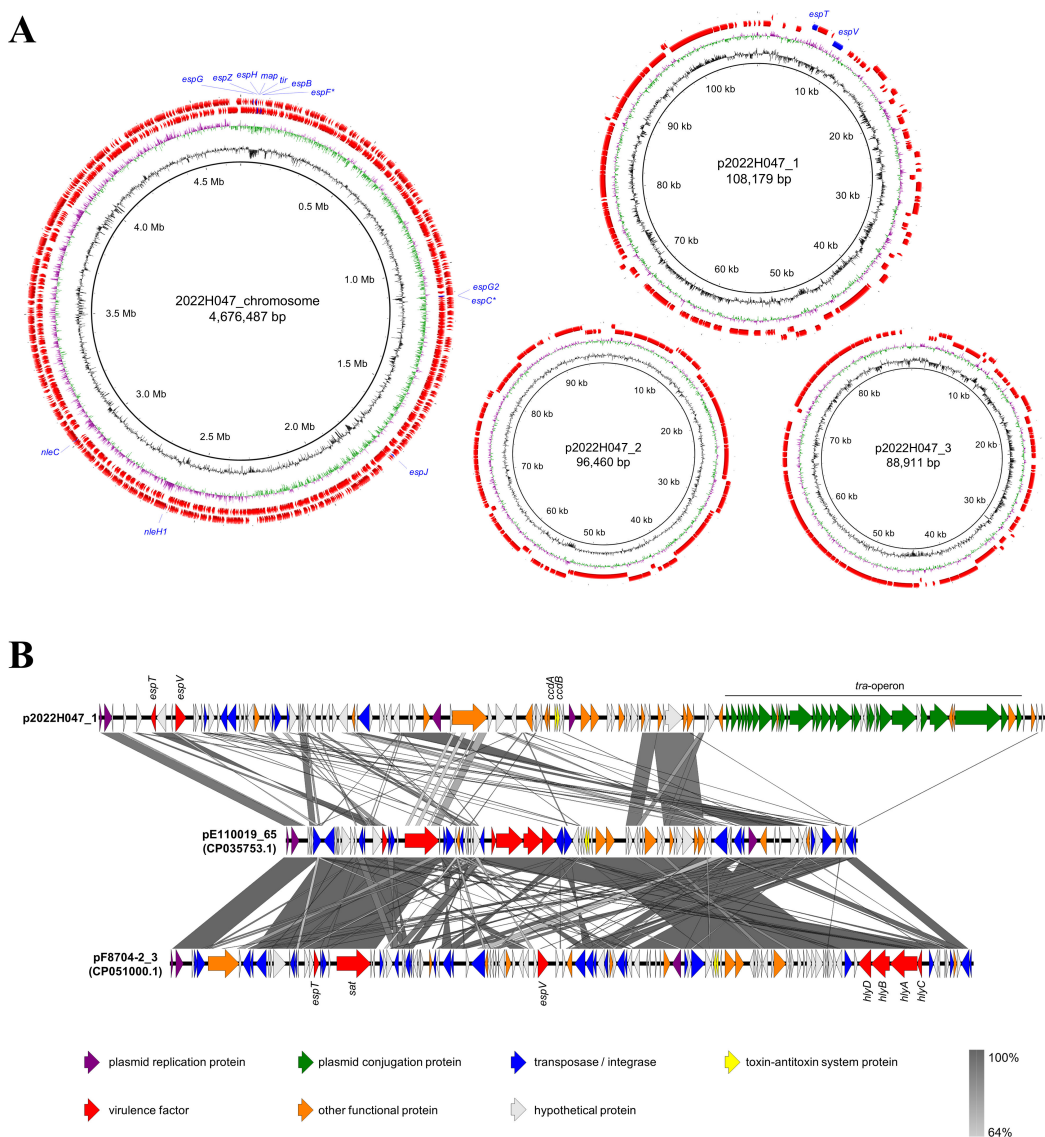
(**A**) Circular map of the 2022H047 genome. Each ring represents GC contents, GC skew, CDS (clockwise), and CDS (counterclockwise), from innermost to outermost, respectively. The genes encoding for effectors of T3SS are highlighted as blue arrows. The pseudogenes are represented by their gene name with an asterisk. GC skew+, green; GC skew−, purple. (**B**) Comparison of *espT*-carrying plasmid sequences. Complete plasmid sequences of 2022H047, E110019 (aEPEC O111:H9), and F8704-2 (aEPECO39:HNM) were compared by BLASTN search. The BLAST identity scale is displayed at the lower right side.

We further constructed the complete genomes of two representative isolates, 2022H047 and 2022H048, which showed different PFGE patterns, since the draft genome is not suitable for identifying the genomic structure. 2022H047 had 4.68 Mb of chromosome and three plasmids with sizes of 108.2, 96.5, and 88.9 kb, while 2022H048 had 4.68 Mb of chromosome and a 108.2 kb plasmid (Fig. 3A; Fig. S1; Table S1). We found seven LEE-encoded T3SS effector genes (*map*, *tir*, *espG*, *espZ*, *espH*, *espB*, and *espF*) and five non-LEE-encoded T3SS effector genes (*espG2*, *espC*, *espJ*, *nleH1*, and *nleC*) on the chromosome of strains 2022H047 and 2022H048 (Fig. 3A; Fig. S2), although *espF* and *espC* were pseudogenes (Table S2). The non-LEE encoded effector genes were found on mobile genetic elements: *espG2*, *espC*, and *espJ* were on integrative elements, and *nleH1* and *nleC* were on prophages (Fig. S2). The intimin subtype was identified as kappa (Fig. S3). The LEE subtype was identified as ST28: *eae*-18, *tir*-11, *espA*-9, *espB*-5, *espD*-6, *espH*-4, and *espZ*-13 (Fig. S3) (18). Additionally, both 2022H047 and 2022H048 had two T3SS effectors (*espV* and *espT*) on the 108.2 kb plasmid (Fig. 3A; Fig. S1). The genes encoded on the *bfp* operon were also negative by whole genome sequencing. No acquired genes or point mutations related to antimicrobial resistance were found in either the chromosome or plasmids. Read mapping and plasmid replicon typing of draft genomes implied the presence of the 108.2 kb plasmid (p2022H047_1) with IncFIIB/IncFIA/IncFII replicon in all isolates (EC22002, EC22005, EC22013, EC22015, and EC22035), although the 96.5 and 88.9 kb plasmids (p2022H047_2 and p2022H047_3) were absent in several isolates (Table S1; Fig. S4).

EspT is a T3SS effector involved in the invasive phenotype of EPEC and was reported as a virulence factor of E110019, a causative agent in a large aEPEC outbreak that occurred in Finland (19). We then focused on the 108.2 kb plasmid carrying *espT* and compared the nucleotide sequences with other *espT*-positive plasmids (Fig. 3B). As of 27 October 2023, we identified two complete plasmid sequences (pE110019_65, CP035753.1 and pF8704-2_3, CP051000.1) that encode EspT using the National Center for Biotechnology Information (NCBI) nt database. The nucleotide sequence of *espT* in the three plasmids was similar. The EspT of 2022H047 was identical to a previously reported EspT2 (20) and harbored two amino acid substitutions with the archetypal EspT—EspT1—encoded in aEPEC strains E110019 and F8704-2 (Fig. S5). However, the plasmid backbone of p2022H047_1 significantly differed from previously reported plasmids (Fig. 3B). The *tra* operon for plasmid conjugational transfer was found only in p2022H047_1. The genes encoding for the toxin-antitoxin system protein, CcdA-CcdB, were found on the plasmid. The EspV gene was found in a plasmid of strain 2022H047 and F8704-2, whereas E110019 harbored *espV* on the chromosome. EspV of 2022H047 was similar to other EspV encoded in aEPEC E110019, rabbit EPEC E22, and *Citrobacter rodentium* ICC168, with 94%, 90%, and 88% identities at the amino acid level, respectively (Fig. S6). Other virulence-related genes (*sat* and *hlyABCD*) were absent in p2022H047_1.

### Phylogenetic analysis

**Fig 4:**
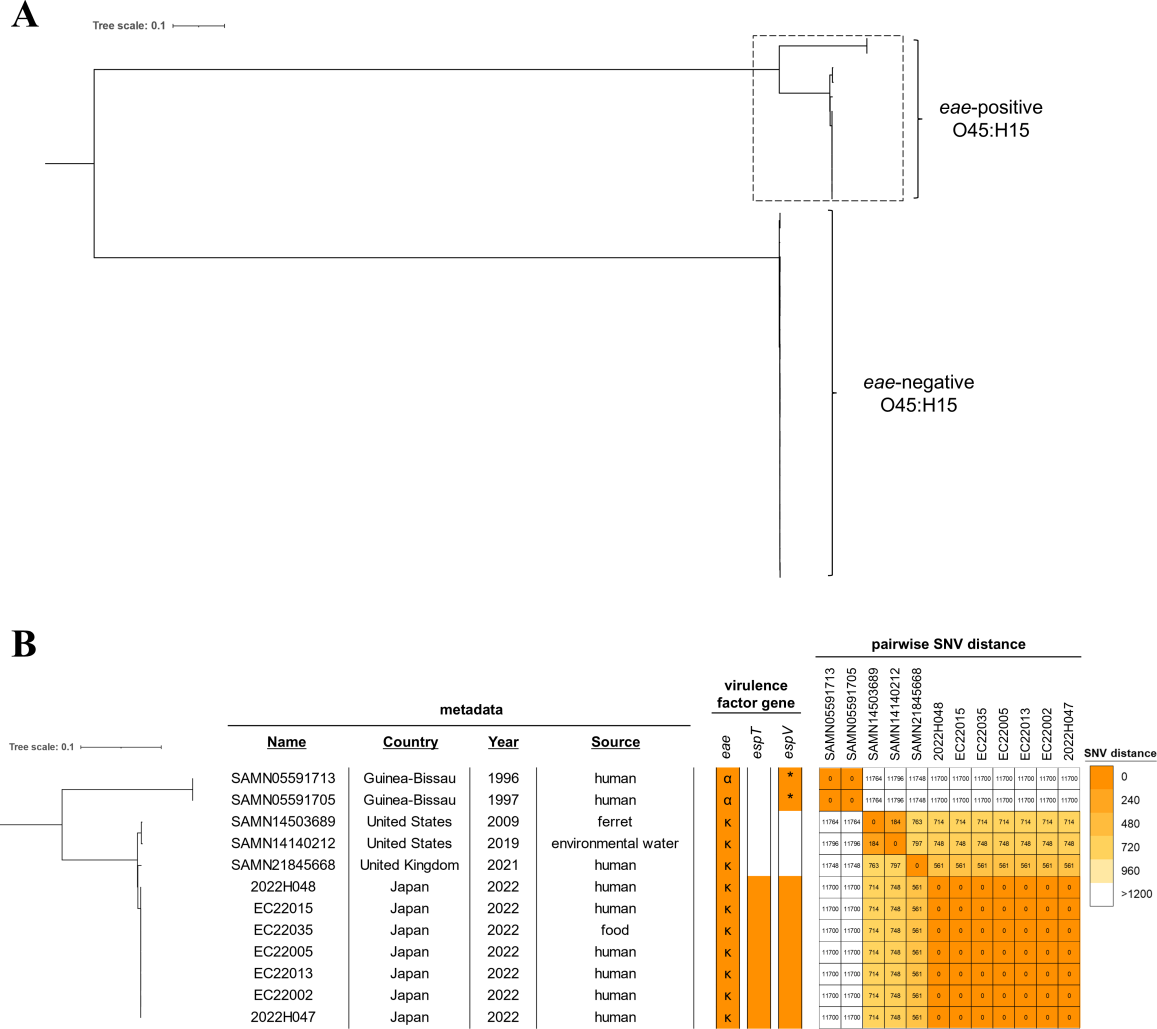
Core-genome SNV-based phylogenetic tree of O45:H15 genomes. (**A**) Maximum likelihood phylogenetic tree of *E. coli* O45:H15 genomes. The dotted square indicates *eae*-positive strain genomes detailed in panel B. (**B**) Subtree of EPEC O45:H15 strains. Metadata, virulence factor gene profile, and number of pairwise SNVs are indicated on the right side of the tree. Virulence factor genes present in the genomes are represented as orange-colored filled boxes, while genes absent in the genomes are represented as open boxes. α and κ represent their intimin subtypes, and the asterisk represents the gene encoding the truncated protein.

We downloaded 31 publicly available *E. coli* genomes whose serotypes were predicted as O45:H15 (Table S3) and conducted a phylogenetic analysis with our EPEC O45:H15 genomes to estimate the origin of our outbreak-related strains. The maximum-likelihood tree of O45:H15 was largely divided into two clusters (Fig. 4A). One was composed of *eae*-positive strains (EPEC O45:H15), while the others contained *eae*-negative strains (non-EPEC O45:H15). EPEC O45 strains were further divided into two groups (Fig. 4B), one carrying intimin-type alpha and the other carrying intimin-type kappa. *espT* was found in our seven sequenced genomes only. *espV* was found both in our sequenced genomes and in the genomes of strains SAMN05591705 and SAMN05591713, although a 33-amino acid N-terminal truncation of EspV was found in SAMN0551705 and SAMN05591713. The number of pairwise SNV distances in our food poisoning-related strains versus other EPEC O45:H15 strains ranged from 561 to 11,700. The most closely related strain was isolated in 2021 in the United Kingdom from a human whose health status was unknown.

To further understand the phylogenetic positions of the isolates, we compared our genomes with those of a wide range of *E. coli* populations. First, we searched *E. coli* genomes based on the MLST scheme using the Enterobase database (21). There was only one ST (ST10782) which was within three of seven allele differences with ST8259 (Fig. S7A). All ST8259 and ST10782 genomes were predicted as serotype O45:H15. Next, we downloaded 362 *E. coli* and *Shigella* genomes, including 250 aEPEC genomes (18), and conducted phylogenetic analysis with EPEC O45:H15 genomes (Fig. S7B; Table S4). The phylogenetic tree identified a distinct clustering of EPEC O45:H15 from other aEPEC isolates. *espT* was detected in 4.1% of database genomes and found across multiple lineages (Fig. S7B).

## DISCUSSION

Originally, EPEC was a certain group of *E. coli* strains that belonged to specific O serotypes (6). Although these classical O-serotype EPEC strains have contributed to several diarrheal outbreaks (12, 16), numerous reports support the notion that non-classical O-serotype EPEC also has the potential to cause outbreaks (13–15). In the current study, we identified aEPEC O45:H15 from feces and food samples as the etiological agent of a foodborne outbreak. One likely explanation for the origin of aEPEC is that aEPEC is one of the variants of Shiga toxin-producing *Escherichia coli* (STEC) that lost stx-converting phages (6). Given that none of the O45:H15 strains downloaded from the database harbored *stx*, it seems unlikely that our aEPEC O45:H15 strains originate from STEC. EPEC O45 has also been reported as one of the serotypes associated with swine postweaning diarrhea (22), although the pathogenicity of this serotype in humans is unclear. Further studies, such as whole genome sequencing of EPEC O45 isolates from swine, may reveal the origin of our outbreak-related strains.

During laboratory identification, bacterial serotyping helps discriminate outbreak-related *E. coli* isolates from commensal ones. In the current case, however, commercial antisera purchased from a Japanese supplier, Denka, did not contain antiserum against O45 antigen, since O45 is a minor *E. coli* serogroup in Japan. Iguchi et al. recently established PCR-based O-typing, called O-genotyping PCR, that covers almost all O serogroups (23). Although PFGE or whole genome sequencing enables strain typing with a higher level of discrimination, the PCR-based approach still has advantages with regard to its speed and cost-effectiveness. In fact, O-genotyping PCR revealed that the same O-serogroup *E. coli* isolates had been recovered from multiple samples in the early stage of the outbreak investigation. The current case exemplifies well the utility of O-genotyping PCR as a rapid and powerful tool to discriminate isolates during laboratory investigation.

Investigations by healthcare centers revealed that the restaurant normally prepared more than 500 meals each day with a few food handlers and that the kitchen of the restaurant was very disorganized. In addition, one of the aEPEC O45:H15-positive employees was found to have been engaged in boiling tofu, an ingredient of the mashed tofu salad with spinach, and had eaten mashed tofu salad with spinach on 13 June, before fecal sampling. It was unclear whether the employee was infected with aEPEC O45:H15 from the mashed tofu salad with spinach or was an aEPEC O45:H15 carrier. The other positive employee was not engaged in preparing lunch boxes between 11 and 13 June. However, since we could not further investigate the contamination source of aEPEC O45:H15, the contamination route—whether employees or other factors such as raw materials—remains unclear.

Bulgin et al. found that EspT modulates host cell cytoskeleton to allow invasion within non-phagocytic cells and proposed a new category of EPEC called invasive EPEC (19). One well-known invasive EPEC strain is E110019, the causative agent of a large outbreak affecting more than 600 pupils as well as 39 adults in Finland (12). To our knowledge, the current foodborne outbreak is the second case of an outbreak by an aEPEC strain harboring *espT*. Although it remains unclear whether the invasive phenotype affects the onset of diarrhea, our case strengthens the hypothesis that the invasive EPEC pathotype has the potential to cause large outbreaks. In contrast to EspT, the role of EspV in infection is less understood. Arbeloa et al. demonstrated that ectopic expression of EspV induced morphological change in host cells. However, an *espV* mutant *C. rodentium* strain showed levels of colonization which were comparable with the wild-type strain in an animal infection model (24). They also reported that wild-type aEPEC E110019 and the *C. rodentium* strain did not induce the morphological change seen in ectopic expression. Although a synergistic effect of EspV with EspT or other effector proteins should be considered, we believe that EspV is unlikely to be a major virulence determinant of this strain.

It is noteworthy that p2022H047_1 contained the *tra* operon, which is required for the conjugative transfer of plasmids (25), suggesting that p2022H047_1 has the potential to transfer horizontally. No other EPEC O45:H15 genome harbored *espT* (Fig. 4B), implying that our isolates acquired *espT* by horizontal gene transfer. The finding of phylogenetic analysis that *espT* was distributed across multiple lineages (Fig. S7B) also enhances the importance of horizontal gene transfer for the movement of *espT*. Furthermore, the gene for the toxin-antitoxin system, *ccdA-ccdB*, enhances the stability of the plasmid (26). Although some of the sequenced genomes lost either or both p2022H047_2 and p2022H047_3, p2022H047_1 was identified in all isolates (Fig. S4). This trait is advantageous for aEPEC O45:H15 in that it allows it to retain a virulence plasmid harboring *espT* toward daughter cells.

Phylogenetic analysis found *espT* in only 4.1% of genomes (Fig. S7B). Arbeloa et al. also reported a low prevalence of *espT* among EHEC, tEPEC, and aEPEC isolates: 0%, 0.8%, and 2%, respectively (20). One notable point of this outbreak is that the age distribution of patients included adults and was not restricted to infants or toddlers (Table 1). Given that E110019 harboring *espT* also infected adult persons (12), invasive EPEC strains may have the potential to cause disease in a wide range of age groups. However, there are no other reports of this rare pathogen, except for E110019 and our isolates, and further outbreak descriptions with whole genome sequence data are required to reveal the pathogenesis of invasive EPEC.

In conclusion, we identified aEPEC O45:H15 as the etiological agent of a foodborne outbreak that occurred in 2022 in Japan. Whole genome sequencing of isolates revealed the presence of a plasmid carrying the T3SS effector genes *espT* and *espV*, whose backbone differed from previously sequenced plasmids. To our knowledge, there have been no reports of food poisoning cases caused by atypical EPEC O45:H15 harboring *espT*. Although several outbreaks of aEPEC have been reported, the genetic background of outbreak-related strains remains unclear. Further genomic analysis of outbreak strains is required to reveal the genetic determinants of the pathogenicity of aEPEC.

## MATERIALS AND METHODS

### Sampling

The outbreak investigation was performed as part of the regional public health services based on the Food Sanitation Act in Japan. Nine hundred thirty-four people consumed the lunch box in total, and 251 of them answered the questionnaires by local health agencies. Fecal samples were collected from 44 patients who lived in Osaka and Hyogo prefectures. In addition, 10 fecal samples of employees, 10 environmental swabs, and 6 food samples were also collected from the restaurant.

### Bacterial isolation and identification

Since the current outbreak occurred across multiple prefectures, laboratory investigations were performed in several local laboratories, namely, the Osaka Institute of Public Health, Hyogo Prefectural Institute of Public Health Science, and Hyogo Prefectural Health and Welfare Offices.

In the Osaka Institute of Public Health, diarrheagenic *E. coli* isolation was performed as described elsewhere (27) using two modified multiplex PCR protocols. These PCR amplifications were used to detect diarrheagenic *E. coli* virulence genes: intimin gene (*eae*), heat-labile enterotoxin gene (LT gene), the gene coding a transcriptional activator of aggregative adherence fimbria I expression in enteroaggregative *E. coli* (*aggR*), heat-stable enterotoxin genes (STh and STp gene), Shiga toxin gene (*stx1* and *stx2*), and cytolethal distending toxin subunit B (Table S5). The PCR reaction mixtures were prepared using a QIAGEN Multiplex PCR Plus Kit (QIAGEN, Hilden, Germany) containing each primer shown in Table S5 and 1 μL DNA template in a total volume of 15 µL. Additionally, modified *Escherichia coli* (EC) broth (Shimadzu Diagnostics, Tokyo, Japan) was used as an enrichment broth for the recovery of *E. coli* from fecal specimens. Fecal samples were inoculated into 10 mL of modified EC and incubated at 36°C for 20 h. A DNA template for PCR to detect diarrheagenic *E. coli* virulence genes was prepared from 100 µL of each modified EC culture by the alkaline lysis method. Briefly, the enrichment culture was centrifuged at 15,000 rpm for 3 min, and the pellet was suspended in 100 µL of 25 mM NaOH. After heat treatment at 95°C for 5 min, the suspension was neutralized with 8 µL of 1 M Tris-HCl (pH 7.5). Centrifuged at 15,000 rpm for 3 min, the supernatant was tested for the two multiplex PCRs described above. PCR-positive cultures were spread-plated on deoxycholate hydrogen sulfide lactose agar (DHL) plates (Shimadzu Diagnostics), and bacterial isolation was performed. O:H serotyping was performed using *E. coli* O-genotyping PCR, *E. coli* H-genotyping PCR (23, 28), and O and H antisera (Denka; Serum Status Institute, Copenhagen, Denmark). Additionally, PCR assay to detect *bfpA* was also performed against *eae*-positive isolates using a single primer pair, EP-1/EP-2 (29). PCR reaction mixture for *bfpA* was prepared using Takara EX Taq Hot Start Version (Takara Bio, Shiga, Japan) containing 5 pmol of each primer and DNA template to a total volume of 12.5 µL. Amplification conditions involved an initial denaturation at 94°C for 5 min, 30 cycles of denaturation at 94°C for 30 s, annealing at 55°C for 30 s, and extension at 72°C for 30 s, followed by a final elongation step at 72°C for 5 min.

At the Hyogo Prefectural Institute of Public Health Science and Hyogo Prefectural Health and Welfare Offices, because *eae*-positive *E. coli* O45 had been previously detected at the Osaka Institute of Public Health, isolation of diarrheagenic *E. coli* was performed with a primary focus on EPEC. Briefly, fecal specimens were spread-plated on MacConkey agar plates (Shimadzu Diagnostics) and incubated at 35°C for 18 h. Suspected *E. coli* cells from either areas of confluent growth or individual colonies on agar plates were transferred to 0.2 mL of Tris-EDTA buffer at pH 8.0 with 5% Chelex 100 Resin (Bio-Rad, Hercules, CA, USA). Resulting cell suspensions were heated at 100°C for 10 min and centrifuged at 10,000 rpm for 10 min, and DNA in the supernatant was analyzed by PCR. PCR reaction mixtures were prepared using Go Taq Green Master Mix (Promega, Tokyo, Japan) containing the primers and 2.5 µL amounts of DNA template in a final volume of 25 µL. The amplification conditions were 94°C for 5 min, followed by 30 cycles of 94°C for 30 s, 55°C for 30 s and 72°C for 1 min, followed by 72°C for 10 min. Amplified products were electrophoresed on a 2.0% (wt/vol) agarose gel and visualized by staining with gel red (Biotium, Fremont, CA, USA) and viewed under UV light. Suspected *E. coli* colonies were picked from plates that were *eae* gene positive on PCR assay and confirmed to be EPEC using conventional biochemical tests and two multiplex PCRs (ExEC and EpAll) to detect 11 diarrheagenic *E. coli* virulence genes (ExEC: *elt*, *estA1*, *estA2*, *stx1*, *stx2*, *stx2f*, and *invE*; EpAll: *eae*, *aggR*, *astA*, and *afaD*) (30, 31). PCR reaction mixtures were prepared using Go Taq Green Master Mix HS (Promega) containing the primers and 3 µL amounts of DNA template in a final volume of 25 µL. The amplification conditions were 94°C for 5 min, followed by 30 cycles of 94°C for 30 s, 50°C (for EpAll, 55°C) for 30 s and 72°C for 1 min, followed by 72°C for 10 min. Stored food samples were homogenized in 9 vol of saline, plated onto DHL plates (Shimadzu Diagnostics), and incubated at 35°C for 24 h. Suspected *E. coli* colonies were picked from the plates and confirmed to be EPEC by ExEC and EpAll PCR. Additionally, the *E. coli* isolates were O:H-serotyped using antisera (Denka), *E. coli* O-genotyping PCR, and *E. coli* H-genotyping PCR (23, 28).

### Antimicrobial susceptibility testing

Antimicrobial susceptibility testing was performed using the Kirby-Bauer disk diffusion method in accordance with procedures established by the Clinical and Laboratory Standards Institute (Performance Standards for Antimicrobial Susceptibility Testing, M02-A12 and M100-Ed31). A total of 17 antibiotics (ampicillin, cefotaxime, gentamicin, kanamycin, imipenem, norfloxacin, ciprofloxacin, nalidixic acid, trimethoprim-sulfamethoxazole, meropenem, ceftazidime, fosfomycin, chloramphenicol, cefoxitin, amikacin, streptomycin, and tetracycline) were tested using the Sensi-disc (Becton Dickinson, Franklin Lakes, NJ, USA).

### PFGE

Molecular typing of EPEC O45:H15 isolates was performed using PFGE with restriction endonuclease XbaI according to the standardized protocols used by PulseNet laboratories (32). The PFGE standard strain *Salmonella enterica* serovar Braenderup H9812 was obtained from the PulseNet program, Enteric Diseases Laboratory Branch, Centers for Disease Control and Prevention (Atlanta, Georgia, USA), through the National Institute of Infectious Diseases (Tokyo, Japan). PFGE was performed on a 1.0% SeaKem Gold Agarose gel (Cambrex Bio Science, Rockland, ME, USA) in 0.5× Tris-borate-EDTA buffer at 14°C and 200 V using a CHEF-DRIII apparatus (Bio-Rad). A linearly ramped switching time of 2.2–54.2 s was applied for 19 h. The gels were stained with ethidium bromide or GelRed, destained, and photographed under UV light. PFGE patterns were compared on dendrograms generated in BioNumerics (Applied Maths, Kortrijk, Belgium) using a UPGMA clustering algorithm and a Dice similarity coefficient with a 1% band matching criterion.

### Whole genome sequencing and gene annotations

The DNA library was prepared using a QIAseq FX DNA Library kit (QIAGEN) or Nextera XT DNA preparation kit (Illumina, San Diego, CA, USA) and sequenced using Illumina sequencer MiSeq or iSeq100 with 150 bp paired-end sequencing. After quality trimming of the sequenced reads with fastp (33), *de novo* assembly was performed using SKESA (34).

Two isolates (2022H047 and 2022H048) were also sequenced with MinION (Oxford Nanopore, Oxford, United Kingdom) using a Rapid Barcoding Kit and a FLO-MIN106-R9.4 flow cell (Oxford Nanopore). Basecalling was performed using Guppy version 6.1.7 with the “super-accurate basecalling” option, and hybrid assembly was performed using Unicycler (35). Assembled genomes were further polished using Pilon (36) with Illumina read data to obtain complete genomes. Dfast-core was used for gene annotation with the use-prodigal option (37).

Clermont typing was performed using a Python script, EzClermont (https://github.com/nickp60/EzClermont), and *in silico* serotyping was performed using ECTyper (38). Identification of virulence factor genes was conducted using the Virulence Factor Database by a BLASTN search with a threshold of 85% identity and 85% coverage. Circular maps were generated using BRIG (39).

### Plasmid comparison

The plasmid that encodes EspT was searched for by TBLASTN search against the NCBI nt database. An archetypal sequence of EspT (CAX32470.1) was used as a query sequence, and complete plasmid sequences which carried EspT with more than 85% identity were used for further analysis. Easyfig (40) was used for visualization.

### Phylogenetic analysis

A chromosome of strain 2022H047 was used as a reference genome. The *E. coli* genomes whose serotypes were predicted as O45:H15 were searched using the Enterobase database (21). SNV calling was performed using bactsnp (41). SNVs located on the prophage region and recombination region were removed from analysis using PHASTEST and gubbins, respectively (42, 43). The maximum likelihood phylogenetic tree based on a general time reversible model with gamma distribution was constructed using IQ-TREE (44) and visualized using iTOL (45). Snippy (https://github.com/tseemann/snippy) and the CFSAN pipeline (46) were also used to confirm the results obtained by the bactsnp pipeline.

The core-gene SNV-based phylogenetic tree was constructed as follows. A wide range of *E. coli* genomes (18) were downloaded from the NCBI database (Table S4) and checked for quality using CheckM (47). The genomes with ≥95% completeness and ≤5% contamination were used for further analysis. Panaroo (48) was used to identify the core genes of the data set genomes, and VeryFastTree (49) was used for tree construction.

## Data Availability

The raw sequence reads and assembled genome contigs were deposited in GenBank/EMBL/DDBJ under the BioProject accession number PRJDB17431 (https://www.ncbi.nlm.nih.gov/bioproject ) (Table S1).

## SUPPLEMENTAL MATERIAL

Supplemental figures aem.00123-25-s0001.pdf Figures S1 to S7.

Supplemental tables aem.00123-25-s0002.xlsx Tables S1 to S5.

ASM does not own the copyrights to Supplemental Material that may be linked to, or accessed through, an article. The authors have granted ASM a non-exclusive, world-wide license to publish the Supplemental Material files. Please contact the corresponding author directly for reuse.
