# Supplementary material for: A foodborne outbreak caused by atypical enteropathogenic Escherichia coli O45:H15 in the Kinki region of Japan
Source: Appl Environ Microbiol. 2025 May 27;91(6):e00123-25. doi: 10.1128/aem.00123-25 (PMC12175500; doi:10.1128/aem.00123-25)
Supplement: Supplemental figures — Figures S1 to S7. [file aem.00123-25-s0001.pdf]

Supplementary Figure 1

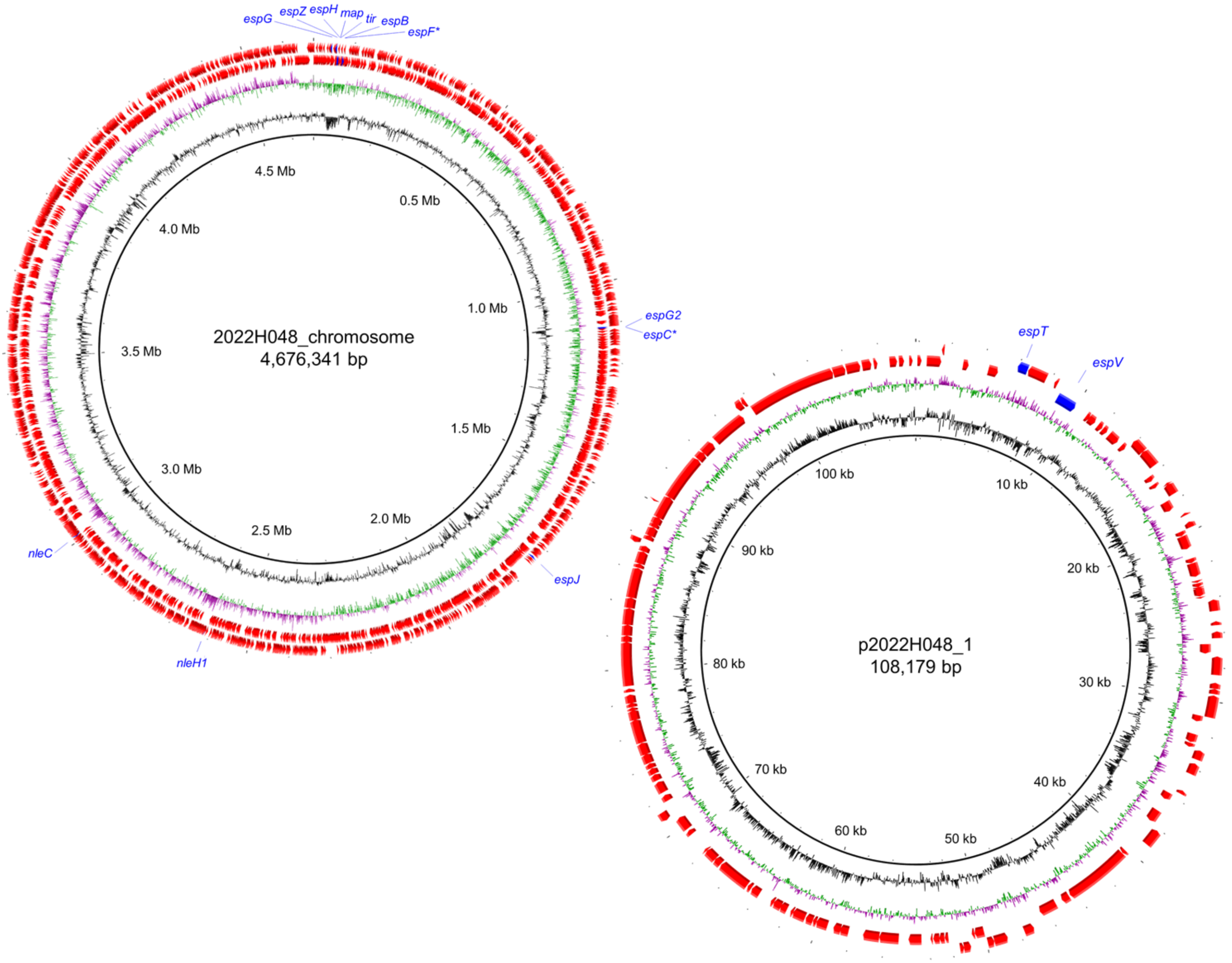

Fig S1. Circular map of strain 2022H048 genome. Each ring represents GC contents, GC Skew, CDS (clockwise), and CDS (counter-clockwise), from innermost to outermost, respectively. Genes encoding for effectors of T3SS are highlighted as blue arrows. Pseudogenes are represented by their gene name with an asterisk. GC Skew+, green; GC Skew-, purple.

Supplementary Figure 2

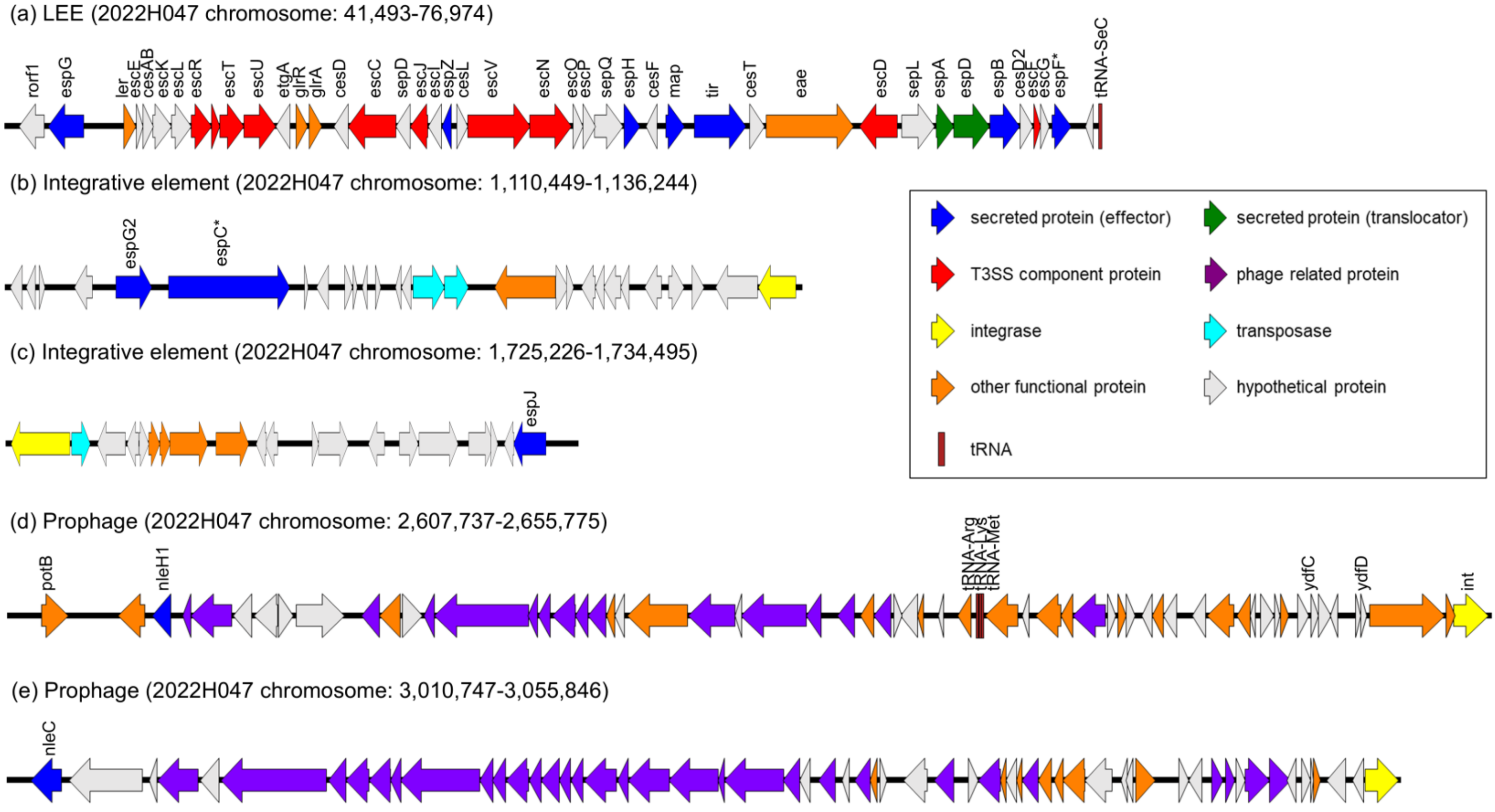

Fig S2. Location and genetic structure of chromosomal encoded effector genes. Pseudogenes are represented by their gene name with an asterisk.

Supplementary Figure 3

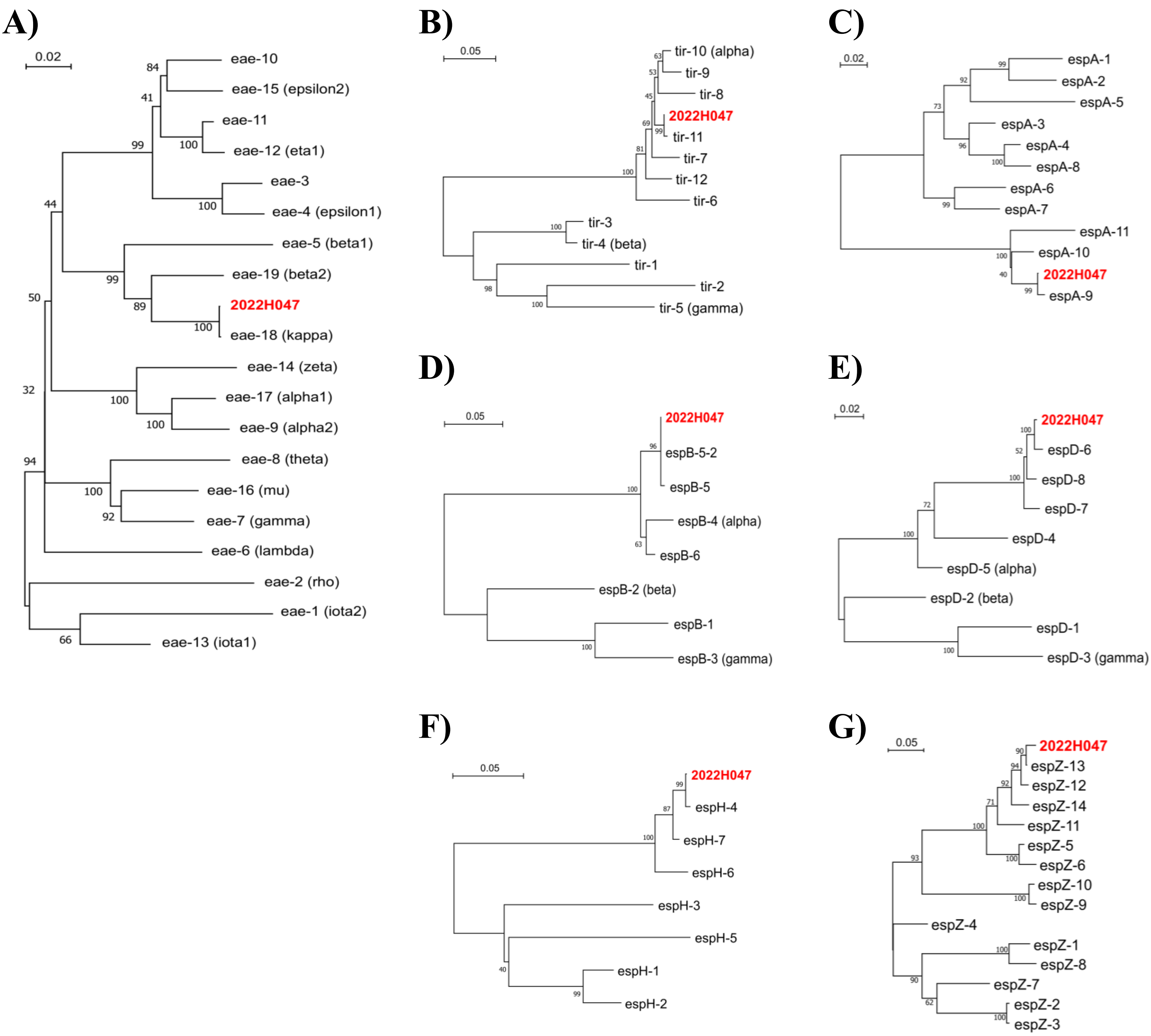

Fig S3. Neighbor-Joining tree of LEE encoded genes. A) *eae*, B) *tir*, C) *espA*, D) *espB*, E) *espD*, F) *espH*, G) *espZ*. Reference sequences were retrieved from a pre-existing database (https://github.com/katholt/srst2).

Supplementary Figure 4

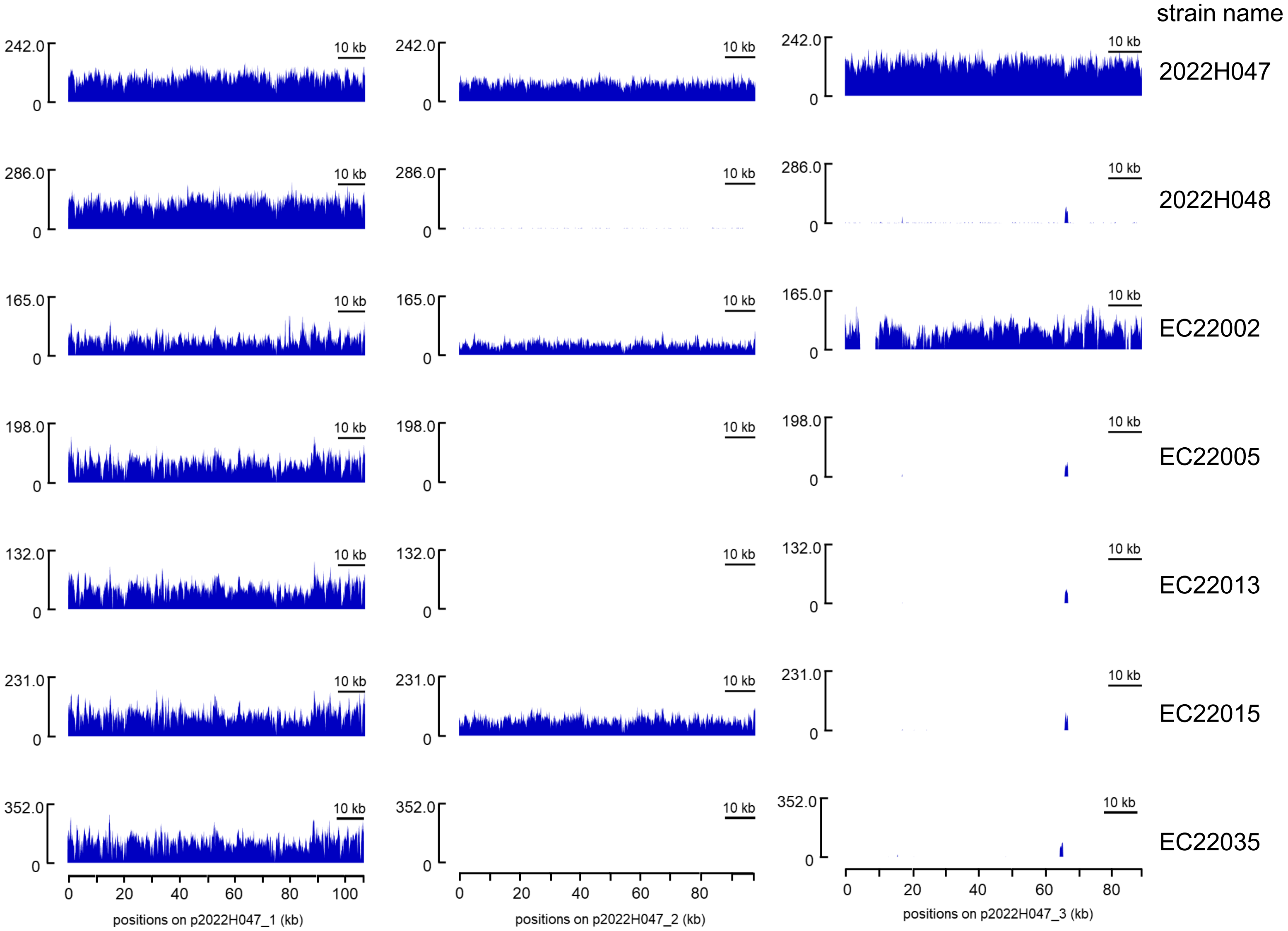

Fig S4. Coverage plot of mapped short read data. The short read sequence data are mapped to each plasmid sequence of strain 2022H047 (p2022H047_1, p2022H047_2, p2022H047_3) and the coverages are shown as a graph. The x-axis represents the positions on each plasmid (kb), and the y-axis represents the coverage.

Supplementary Figure 5

A)

```
                                                                        *
E110019(EspT1) MPGTVNSCGFGFSITKAPYSSGQKPVIDGFFLGARRISFSYPRLESELIQCINLKNEGKK    60
F8704-2        MPGTVNSCGFGFSITKAPYSSGQKPVIDGFFLGARRISFSYPRLESELIQCINLKNEGKK    60
2022H047       MPGTVNSCGFGFSITKAPYSSGQKPVIDGFFLGARRISFSYPRLESELIQCINLKNEGKK    60
EspT2          ---TVNSCGFGFSITKAPYSSGQKPVIDGFFLGARRISFSYPRLESELIQCINLKNEGKK    57
EspT3          ---TVNSCGFGFSITKAPYSSGQKPFIDGFFLGARKISFSYPRLESELIQCINLKNEGKK    57
```

```
                                                                        *
E110019(EspT1) NEWMKEESICFVSRDVNKLLDMFAKNNQTNIPKGVRERVFQLASFYCGFSLDARCAQAST    120
F8704-2        NEWMKEESICFVSRDVNKLLDMFAKNNQTNIPKGVRERVFQLASFYCGFSLDARCAQAST    120
2022H047       NEWMKEESICFVSRDVNKLLDMFAKNNQTNIPKGVRERVFQLASFYCGFSLDARCAQTST    120
EspT2          NEWMKEESICFVSRDVNKLLDMFAKNNQTNIPKGVRERVFQLASFYCGFSLDARCAQTST    117
EspT3          NEWMKEEGICFVSRDVNKLLDMFAKNNQTNIPEGVRERVFQLASFYCGFSLDARCAQTST    117

E110019(EspT1) HHMILNSQYFQKKMDTLLTSVDINVRNQCVRTALSSLADTFFENNVNNIDMNKLRDRVHN    180
F8704-2        HHMILNSQYFQKKMDTLLTSVDINVRNQCVRTALSSLADTFFENNVNNIDMNKLRDRVHN    180
2022H047       HHMILNSQYFQKKMDTLLTSVDINVRNQCVRTALSSLADTFFENNVNNIDMNKLRDRVHN    180
EspT2          HHMILNSQYFQKKMDTLLTSVDINVRNQCVRTALSSLADTFFENNVNNIDMNKLRDRVHN    177
EspT3          HHMILNSQYFQKKMDTLLTSVDINVRNQCVRTALSSLADTFFENNVNNIDMNKFRDRVHN    177

                          *
E110019(EspT1) TIVQEVQRNLKYV    193
F8704-2        TIVQEVQRNLKYV    193
2022H047       TIVQEVQRNLKCV    193
EspT2          TIVQEV-------    183
EspT3          TIVQEV-------    183
```

B)

identity (upper right) / distance (lower left)

	E110019	F8704-2	2022H047	EspT2	EspT3
E110019		1.00	0.99	0.99	0.97
F8704-2	0		0.99	0.99	0.97
2022H047	2	2		1.00	0.97
EspT2	1	1	0		0.97
EspT3	6	6	5	5	

Fig S5. Comparison of amino acid sequences of EspT. (A) Result of multiple alignments of EspT sequences. Amino acid mutation sites are highlighted with a gray background. Asterisks indicate amino acid residues that differ between archetypal EspT1 and EspT of 2022H047. (B) Amino acid differences among each EspT sequence. The number of residues that differ between each EspT sequence are shown at the lower left side of the table, while sequence identity is shown on the upper right side of the table. Accession number of each sequence are as follows: E110019 (aEPEC O111:H9), CP035753.1; F8704-2 (aEPEC O39:HNM), CP051000.1; EspT2 (aEPEC O2:H49), FM992862.1;EspT3 (aEPEC O104:H2), FM992864.1.

Supplementary Figure 6

A)

```
2022H047    MFAAKPEISQRCISGFLTTGPNDENANNKPSSDMANRIKLVVIDSQIGIYTTDSNTLKDG 60
E110019     MFAAKPELSQRCISGLLTTGPNDENVNNKPSSDMPNRIKPVVSDSQIGIYTTDSNTLKDG 60
E22         MFAAKPESSQRSISGLFSPRSNGENASNKPSSGMANRIKPVVSDSQIGRYTTDSNTLKDG 60
ICC168      MFAAKPDSSQRTISGFFTAGANDEYANNKPSSIMADRIKLVVRDTQSGRYTTNCNTLKDG 60

2022H047    LCSQAHIGTQPYRPSVGSPLTVRSFLQITLSNNNYKSLEHLQTRAENYLRHRKAEENILR 120
E110019     LCSQAHIGTQPYRPGADSPITVRSFLQITLSNNNYKSLEHLQTRAENYLRYRKAEENILR 120
E22         LCSQAHIGTQPYRPGADSPLTVRSFLQITLSNNNYKSLEHLQTRAENYLRHRKAEEKILR 120
ICC168      LYSQAHIGTQPYLPGADSPLTVRTFLQITLSNNNYKSFEHLQTRAENYLRHRNAEDKLLR 120

2022H047    STVEGLTNPESPVFKQTAWMGHLERGLWKTETRWDGNDREQLGKEALGSEEPKPGSPFYG 180
E110019     STVEGLTNPESPVFKQTAWMGHLERGLWKTETRWDGNDREQLGKEALGSEEPKPGSPFYG 180
E22         STVEGLTNPESPVFKQAAWMGHLERGLWKTETRWNGNDRDQLGKEALGSEEPKPGSPFYG 180
ICC168      ATVEGLTNPESPVFKQTAWMGHLERGLWKTEAHWDGNDREQLGNEALGSEEPKPGSPFYG 180

2022H047    SRGLKLSDSARSAFSMMLYGSEGPFTKEQALSGFELAQTGQVLAGRLKIQERVKFRADNR 240
E110019     SRGLKLSDSAHSAFSMMLCGSEGPFTKEQALSGFELAQTGQVLAGRLKIQERVKFRADNR 240
E22         SRGLKLSDSARSAFSMMLCGSAGPFTKEQTLSGFELAQTGQVLAGRLKIQERMTFRANNR 240
ICC168      SRGLKLSDSARSAFSMMLFGSAGPFTKEQAISGFELAQTGQVLAGRLKIQERVKFREDNR 240

2022H047    IDAQRNGTHSTRTPTGMDLSQDIGTIMRDQAGLPVMSGTSGSSSDAALATRYAAEYFGKT 300
E110019     IDAQRNGTHSTRTPTGMDLSQDIGTIMRDKAGLPVMSGTSGSSSDATLATRYAAEHFGKT 300
E22         VDAQRNGTHSTRTQTGMDLSQDIGTIMRDKAGLPVMSGTSGSSSDATLATRYAAEHFGKT 300
ICC168      VDAQRNGTHSTRTQTGMDLSHDIGTIMRDKAGLPVMSGTSGSSSDAALATRYAAEYFGKT 300

2022H047    WTAPGLNQTEGCKAISDLSHHYFRAEGSSPPQSMATGINKVRCDAGMKEKHVNTLDIFTH 360
E110019     WAAPGLSQAEGCKAISDLSHHYFRAEGSSPPQSMATGINKVRYDAGMEEKYVNTLDIFTH 360
E22         WAAPGLSQAEGCKAISDLSHHYFRAEGSSPPQSMATGINKVRCDAGMEEKHVNALDIFTH 360
ICC168      WAAPGLNQSEGCKAISDLSHHYFRAEGSSPPQSMATGINKVRCDAGMEEKHVNTLDIFTH 360

2022H047    SYPEIYAGVALTIAGASGNDEQAMYNVTQEAAHILHEAETKD     402
E110019     SYPEIYAGVALTIAGAGGNDEQAMYNVTQEAARILHEAETKD     402
E22         SYPEIYAGVALTIAGAGGNDEQAMYNVTQEAARILHEAETKD     402
ICC168      SYPEIYAGVALTLAGARGNDEQVMHNVTQETVRLLREAETKD     402
```

B)

identity

distance

	2022H047	E110019	E22	ICC168
2022H047		0.94	0.90	0.88
E110019	24		0.93	0.86
E22	39	30		0.87
ICC168	50	55	53	

Fig S6. Comparison of amino acid sequences of EspV. (A) Result of multiple alignments of EspV sequences. Amino acid mutation sites are highlighted with a gray background. (B) Amino acid differences among each EspV sequence. Number of residues that differ between each EspV sequence is shown at the lower left side of the table, while sequence identity is shown at the upper right side of the table. Accession number of each sequence is as follows: E110019 (aEPEC O111:H9), CP035753.1; E22 (rabbit EPEC), AAJV00000000; ICC168 (Citrobacter rodentium), FN543502.1.

Supplementary Figure 7

A)

Allele Differences	ST	*adk*	*fumC*	*gyrB*	*icd*	*mdh*	*purA*	*recA*	Serotypes
0	8259	13	14	694	923	16	10	593	O45:H15
1	10782	13	14	694	923	16	834	593	O45:H15
2	not found								
3	not found								

B)

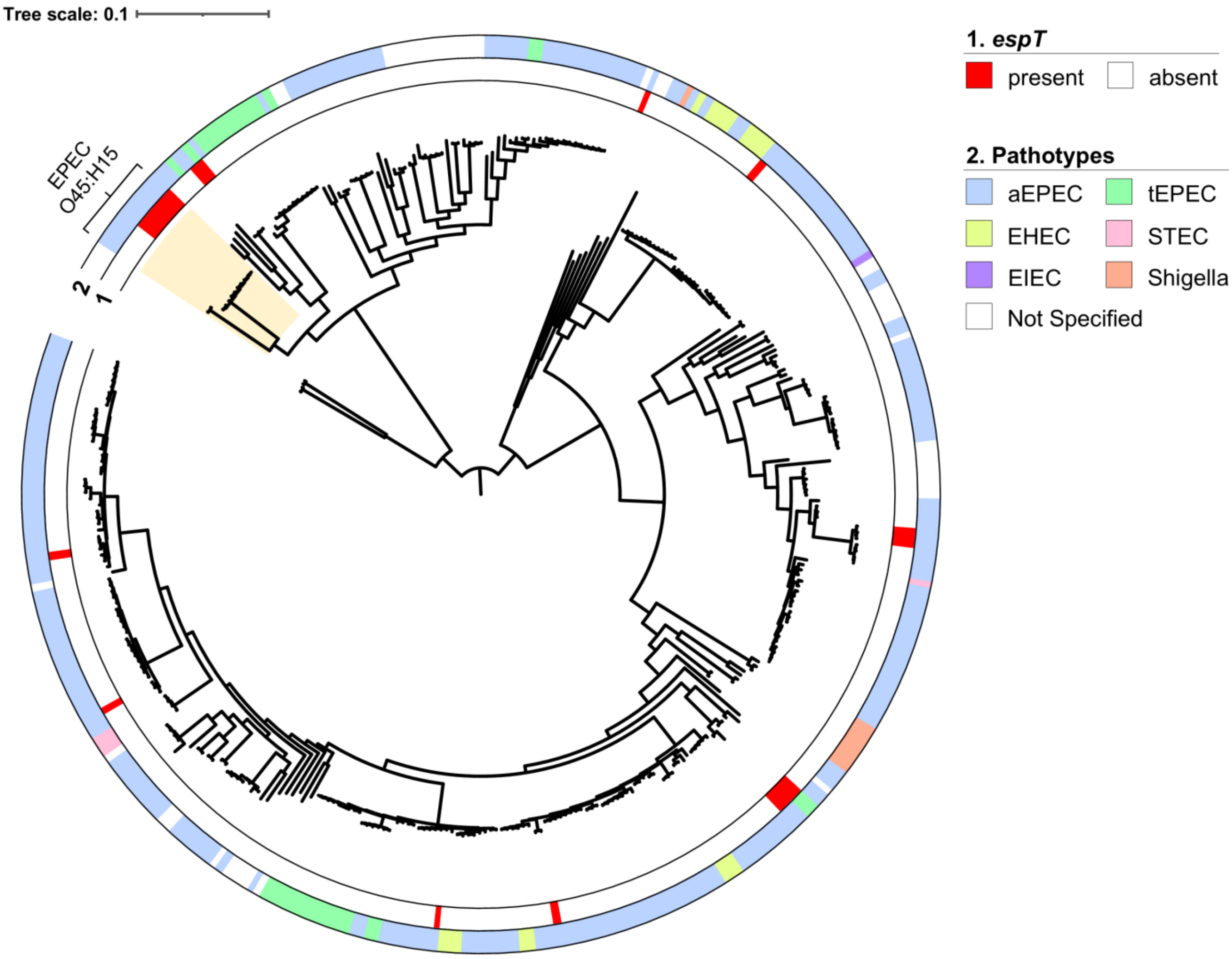

Fig S7. (A) Result of Enterobase search for similar STs with ST8259. STs within 3 allele differences were searched in the database. (B) The phylogenetic tree of EPEC O45:H15 isolates with 367 *E. coli* genomes downloaded from the database. The branch of EPEC O45:H15 genomes is highlighted in light yellow. The presence/absence of *espT* and pathotypes are indicated outside of the tree.
